# Body Fat Percentage Prediction Using Intelligent Hybrid Approaches

**DOI:** 10.1155/2014/383910

**Published:** 2014-03-02

**Authors:** Yuehjen E. Shao

**Affiliations:** Department of Statistics and Information Science, Fu Jen Catholic University, 510, Chung-Cheng Road, Xinzhuang District, New Taipei City 24205, Taiwan

## Abstract

Excess of body fat often leads to obesity. Obesity is typically associated with serious medical diseases, such as cancer, heart disease, and diabetes. Accordingly, knowing the body fat is an extremely important issue since it affects everyone's health. Although there are several ways to measure the body fat percentage (BFP), the accurate methods are often associated with hassle and/or high costs. Traditional single-stage approaches may use certain body measurements or explanatory variables to predict the BFP. Diverging from existing approaches, this study proposes new intelligent hybrid approaches to obtain fewer explanatory variables, and the proposed forecasting models are able to effectively predict the BFP. The proposed hybrid models consist of multiple regression (MR), artificial neural network (ANN), multivariate adaptive regression splines (MARS), and support vector regression (SVR) techniques. The first stage of the modeling includes the use of MR and MARS to obtain fewer but more important sets of explanatory variables. In the second stage, the remaining important variables are served as inputs for the other forecasting methods. A real dataset was used to demonstrate the development of the proposed hybrid models. The prediction results revealed that the proposed hybrid schemes outperformed the typical, single-stage forecasting models.

## 1. Introduction

In recent years, cancer, heart disease, and diabetes have been reported to be the leading causes of death for most countries in the world [1, 2]. One of the most common risk factors for those diseases is the obesity, and excess of body fat often leads to obesity. One common cause of heart disease is the process called atherosclerosis. This happens when fat accumulates in the blood vessels, and it usually results in the thicker walls of the vessels. The thicker walls lead to a reduced flow of blood to the heart, and the heart becomes damaged resulting in a heart attack. It has been reported that excess body fat can increase the risk of six different types of cancers, including bowel, oesophagus, pancreas, kidney, endometrium, and breast cancers, respectively [3]. In addition, type II diabetes is also found in those who are carrying too much body fat. Therefore, how to avoid obesity has become a very important issue.

Although excess of body fat causes obesity, extremely low BFP is also undesirable, as there are minimum requirements for brain function. Accordingly, knowing the BFP can provide a great deal of information regarding the current state of health.

Maintaining a good BFP is a must for human health. However, accurate and convenient ways to measure body fat are not straightforward [4, 5]. For example, the hydrostatic weighing was reported to be a reliable method for the measurement of body fat content, but it is not convenient [4]. A new technology, Dual Energy X-ray Absorptiometry (DEXA), is very accurate and precise to measure the body fat. However, DEXA may suffer from standardization issues; that is, results may vary with the specific equipment manufacturer, data collection methods, and/or software analysis. Consequently, it is desirable to have some convenient methods to predict the BFP.

While some typical studies have employed data mining techniques to classify the existence of certain diseases [2, 6, 7], the present study focuses on the development of intelligent forecasting techniques to effectively predict the BFP. The body fat datasets used in this study were real data obtained from Johnson [5]. The datasets contain the BFP which were determined by underwater weighing and 13 body circumference measurements for 252 people. Although one can employ the aforementioned variables to predict BFP through the use of multiple regression (MR) techniques or some machine learning approaches, the true relationship between these measurements and BFP may not be easy to determine. Several studies have used multiple regression techniques to build a forecasting model to estimate the BFP [5, 8–10]. However, the MR models are criticized for its strong assumptions such as variation homogeneity [2]. In addition, some data mining techniques, such as artificial neural network (ANN), multivariate adaptive regression splines (MARS), and support vector regression (SVR), have become alternatives in modeling forecasting problems due to their capability to capture complex nonlinear relationships among variables [11–13]. Those data mining techniques have been reported to have better forecasting capability than the regression technique [14–18]. Nevertheless, those techniques may have some limitations. For example, ANN has been criticized for its long training process in designing the optimal network's topology. Also, ANN is unable to identify the relative importance of potential input variables [19–22]. Additionally, using a single technology to address all of the prediction problems may not always be possible [23].

To overcome the aforementioned difficulties and maintain the prediction accuracies of existing approaches for BFP, this study is aimed at proposing single and hybrid forecasting models to predict BFP. The single-stage forecasting modeling includes MR, ANN, MARS, and SVR approaches. The hybrid models integrate two modeling components. The first component of the model uses its own feature to capture the important but fewer explanatory variables. The second component of the hybrid schemes generates the predictions based on those explanatory variables. Because MR and MARS have great capability to select the important explanatory variables, in this study, the combinations of MR and ANN (MR-ANN), MR and MARS (MR-MARS), MR and SVR (MR-SVR), MARS and MR (MARS-MR), MARS and ANN (MARS-ANN), and MARS and SVR (MARS-SVR) are employed as the hybrid forecasting models.

In terms of prediction capability, this study compares the typical single-stage forecasting models and the proposed hybrid model for BFP application. The mean absolute percentage error (MAPE), the root mean square error (RMSE), and the mean absolute difference (MAD) are used as the forecasting accuracy measures. The superior prediction capability of the proposed hybrid approach is addressed. The remainder of this study is organized as follows. The following section introduces the methodologies of MR, ANN, MARS, and SVR, and a literature review is also provided. The designed MR, ANN, MARS, and SVR models are presented in Section 3, and a real BFP dataset is used to verify the typical and proposed forecasting models. The performances for all of the forecasting models are demonstrated and discussed. The final section addresses the research findings and concludes this study.

## 2. Research Methodologies

This study considers MR, ANN, MARS, SVR, and their hybrid modeling schemes as possible forecasting models for BFP. These methodologies are addressed as follows.

### 2.1. Multiple Regression Modeling

Multiple regression analysis can be deemed as one of the most used statistical methods in modeling real-world applications. The MR involves setting up the relationships between one response variable and several explanatory variables. The performance of MR is acceptable when the assumptions have been met. However, the assumptions of the MR model may confine its application. The general MR model is represented as follows:

(1) $$\begin{array}{c} y=\beta_{0}+\beta_{1}X_{1}+\beta_{2}X_{2}+\cdots +\beta_{p}X_{p}+\varepsilon , \end{array}$$

where *β*_0_,*β*_1_, *β*_2_,…, *β*_*p*_ are model parameters and *ε* is the error term. The *ε* accounts for the variability in *y* that cannot be explained by the linear effect of the *p* explanatory variables. There are four assumptions about the *ε* in MR model, and they are as follows.

1. The *ε* is a normally distributed random variable.
2. The *ε* is a random variable with a mean value of zero; that is, *E*(*ε*) = 0.
3. The variance of *ε* is denoted by *σ* ^2^ and is the same for all values of the explanatory variables *X*_1_,*X*_2_,…,*X*_*p*_.
4. The values of *ε* are independent.

Also, because collinearity among explanatory variables will lead to imprecise estimates and serious stability problems, the collinearity diagnosis procedure should be performed first before screening important explanatory variables. In this study, a well-known criterion, the variance inflation factor (VIF), is applied to examine collinearity. The VIF is described as follows:

(2) $$\begin{array}{c} \text{VIF}\left(X_{j}\right)=\frac{1}{1-R_{j}^{2}},\;j=1,2,\ldots ,k, \end{array}$$

where *R*_*j*_^2^ is the coefficient of determination of a regression *X*_*j*_ that evaluates all other explanatory variables. The tolerance is defined as the reciprocal of the VIF. It has been suggested that when the value of VIF is greater than 10, the sample set may have enough variation to suggest serious multicollinearity. This study used the technique where one or some explanatory variables could be dropped from the model in order to lessen the collinearity and thus reduce the standard errors of the estimated regression coefficients of the explanatory variables remaining in the model. In addition to the simplicity and effectiveness, this technique has another advantage of reducing the number of explanatory variables. This characteristic is very suitable for hybrid modeling since it usually captures less explanatory variables for the initial stage of modeling.

Typically, when considerable explanatory variables are involved in the MR design, a great amount of computation is required for examining a large volume of computer outputs, much of which are associated with poor MR models. As a consequence, three variable selection procedures are employed in this study. Those three selections include forward selection, backward elimination, and stepwise regression procedures. Given a BFP dataset with 13 explanatory variables, this study uses the aforementioned selection procedures to select the explanatory variables that lead to the best model.

### 2.2. Artificial Neural Network Modeling

Due to ANN's associated memory characteristic and its generalization capability, ANN has been increasingly utilized for modeling nonstationary processes [24–28].

ANN is usually classified into two categories: feedforward and feedback networks [28]. The nodes in the ANN can be divided into three layers: the input, the output, and one or more hidden layers. The output of each neuron in the input layer is the same as the input to that neuron. For each neuron *j* in the hidden layer and neuron *k* in the output layer, the net inputs are given by

(3) $$\begin{array}{c} \text{ne}{\text{t}}_{j}=\sum_{i}w_{ji}\times o_{i},\;\;\text{ne}{\text{t}}_{k}=\sum_{j}w_{kj}\times o_{j}, \end{array}$$

where *i*(*j*) is a neuron in the previous layer, *o*_*i*_(*o*_*j*_) is the output of node *i*(*j*), and *w*_*ji*_(*w*_*kj*_) is the connection weight from neuron *i*(*j*) to neuron *j*(*k*). The neuron outputs are given by

(4) $$\begin{array}{c} o_{i}={\text{net}}_{i}, \end{array}$$

(5) $$\begin{array}{c} o_{j}=\frac{1}{1+\exp^{-({\text{net}}_{j}+\theta_{j})}}=f_{j}\left({\text{net}}_{j},\theta_{j}\right), \end{array}$$

(6) $$\begin{array}{c} o_{k}=\frac{1}{1+\exp^{-({\text{net}}_{k}+\theta_{k})}}=f_{k}\left({\text{net}}_{k},\theta_{k}\right), \end{array}$$

where net_*j*_(net_*k*_) is the input signal from the external source to the node *j*(*k*)  in the input layer and *θ*_*j*_(*θ*_*k*_) is the bias. The transformation function shown in (4) to (6) is called a sigmoid function.

The generalized delta rule is the conventional technique used to derive the connection weights of the feedforward network [28]. Initially, a set of random numbers is assigned to the connection weights. Then, to determine the pattern *p* with a target output vector *t*_*p*_ = [*t*_*p*1_, *t*_*p*2_,…,*t*_*pM*_]^*T*^, the sum of the minimized squared error is given by

(7) $$\begin{array}{c} E_{p}=\frac{1}{2}\sum_{j=1}^{M}{\left(t_{pj}-o_{pj}\right)}^{2}, \end{array}$$

where *M* is the number of output nodes.

### 2.3. Multivariate Adaptive Regression Splines Modeling

MARS has been generally applied in many fields [29–33]. The general MARS function can be represented as follows [33]:

(8) $$\begin{array}{c} \hat{f}\left(x\right)=b_{0}+\sum_{m=1}^{M}b_{m}\prod_{k=1}^{K_{m}}\left[S_{km}\left(x_{\nu (k,m)}-t_{km}\right)\right], \end{array}$$

where *b*_0_ and *b*_*m*_ are parameters, *M* is the number of basis functions (BF), *K*_*m*_ is the number of knots, *S*_*km*_ takes on values of either 1 or −1 and indicates the right or left sense of the associated step function, *ν*(*k*, *m*) is the independent variable, and *t*_*km*_ is the knot location.

A two-stage process is usually used to choose the optimal MARS model. Initially, a large number of basis functions were used to fit the data. Secondly, the basis functions with the least contributions were deleted using generalized cross-validation (GCV) criterion. A measure of variable importance can be obtained by observing the decrease in the calculated GCV values when a variable is removed from the model. The GCV can be expressed as follows:

(9) $$\begin{array}{c} \text{LOF}\left({\hat{f}}_{M}\right)=\text{GCV}\left(M\right)=\frac{\left(1/N\right)\sum_{i=1}^{N}{\left[y_{i}-{\hat{f}}_{M}\left(x_{i}\right)\right]}^{2}}{{\left[1-\left(C\left(M\right)/N\right)\right]}^{2}}. \end{array}$$

### 2.4. Support Vector Regression Modeling

While support vector machine (SVM) is a powerful technique in machine learning areas, SVR can be deemed as a special form of SVMs. Due to its prediction capability, SVR has been used for predictions in many fields [34–36]. Based on the computation of a linear regression function in a high-dimensional feature space, the inputs for SVR are mapped via a nonlinear function. The modeling of SVR can be described as follows. Suppose

(10) $$\begin{array}{c} f\left(x\right)=\left(w\cdot \Phi \left(x\right)\right)+b, \end{array}$$

where *w* is the weight vector, *x* represents the model inputs, *b* is a bias, and Φ(*x*) stands for a kernel function which uses a nonlinear function to transform the nonlinear input to be linear mode in a high-dimensional feature space.

Usually, the regression modeling obtains the coefficients through minimizing the square error, which can be considered as empirical risk based on loss function. The *ε*-insensitivity loss function was introduced [37], and it can be described as follows:

(11) $$\begin{array}{c} L_{\varepsilon }\left(f\left(x\right),y\right)=\{\begin{array}{cc} \left|f\left(x\right)-y\right|-\varepsilon , & \text{if}\;\left|f\left(x\right)-y\right|\geq \varepsilon ,\\ 0, & \text{otherwise,} \end{array} \end{array}$$

where *y* is the target outputs; *ε* defines the region of *ε*-insensitivity. When the predicted value falls into the band area, the loss is zero. However, when the predicted value falls outside the band area, the loss is defined as the difference between the predicted value and the margin.

When empirical risk and structure risk are both considered, the SVR can be set up to minimize the following quadratic programming problem:

(12) Minimize: 12||w||2+C∑i=1n(ξi+ξi∗)subject to (yi−(w·Φ(xi))−b≤ε+ξi(w·Φ(xi))+b−yi≤ε+ξi∗ξi,ξi∗≥0, for  i=1,…,n,

where *i* = 1,…, *n* is the number of training data, (*ξ*_*i*_ + *ξ*_i_*) represents the empirical risk, (1/2)||*w*||^2^ stands for the structure risk preventing overlearning and lack of applied universality, and *C* is a modifying coefficient representing the trade-off between empirical risk and structure risk. With an appropriate modifying coefficient *C*, band area width *ε*, and kernel function, the optimum value of each parameter can be solved by Lagrange procedure.

The SVR-based regression function can be described as follows [37]:

(13) f(x,w)=f(x,r,r∗)=∑i=1N(ri−ri∗)Φ(x,xi)+b,

where *r*_*i*_ and *r*_i_* are Lagrangian multipliers and satisfy the equality *r*_*i*_ *r*_i_* = 0. Additionally, since the radial basis function (RBF) is the most widely used kernel function [36], this study uses it for our experimental study. The RBF can be defined as follows:

(14) $$\begin{array}{c} \phi \left(x_{i},x_{j}\right)=\exp\left(\frac{-{||x_{i}-x_{j}||}^{2}}{2\sigma^{2}}\right), \end{array}$$

where *σ* denotes the width of the RBF.

## 3. BFP Data and Modeling Results

### 3.1. The BFP Dataset

In order to compare the forecasting performance of the single-stage and the proposed hybrid models, a real dataset of BFP is analyzed [5]. The dataset consists of 252 records. Each person consists of 15 variables and they are summarized in Table 1 (i.e., readers can refer to the website http://wiki.stat.ucla.edu/socr/index.php/SOCR_Data_BMI_Regression for more details and descriptions about the dataset).

In this dataset, the response variable BFP is denoted by *Y*. Since the BFP (*Y*) is difficult to measure, Siri's equation, as in (15), is used to compute the value of *Y*:

(15) $$\begin{array}{c} Y=\frac{495}{D}-450. \end{array}$$

In the initial “cleaning” phase for this real dataset, this study has found the body fat measurements of the 96th, 172nd, and 182nd cases are 0.4, 0.7, and −3.6, respectively. Generally speaking, those three measurements deviate too much from normal conditions, and this study has decided to delete those three cases. This study also deleted the 42nd case where the height is only 74.93 cm. Consequently, the sample size becomes 248 cases. With the 248 cases used in this study, the first 174 cases (around 70% of the total cases) were selected as the model training sample while the remaining 74 (around 30% of the total cases) will be retained as the testing sample.

### 3.2. Typical Single-Stage Modeling Approaches

This study considers the BFP (i.e., *Y*) as the response variable and the thirteen body circumference measurements (i.e., *X*_1_  to  *X*_13_) as the explanatory variables. The first variable, density determined from underwater weighing, in Table 1 is just for computing the values of BFP. To exclude variables with high collinearity, the Pearson correlation coefficients between variables are used.  Table 2 shows the corresponding results. When the correlation coefficient *ρ*_*X*_*i*_,*X*_*j*__between variables *X*_*i*_ and *X*_*j*_ is greater than 0.7, we exclude the variable that has a lower relationship with *Y* (i.e., exclude the variable with a smaller correlation coefficient *ρ*_*X*,*Y*_). After discarding variables with high collinearity, eight explanatory variables *X*_1_, *X*_2_, *X*_4_, *X*_6_, *X*_10_, *X*_11_, *X*_12_, and *X*_13_ remain in the MR models.

In addition, this study employed three selection techniques to develop alternative MR models for BFP. These three techniques include forward selection, backward elimination, and the stepwise regression analysis. The forward selection is similar to the stepwise selection. The first explanatory variable is selected for inclusion of the regression equation is the one with the largest positive or negative correlation with the response variable, *Y*. This explanatory variable is entered into the regression equation only if it satisfies the tolerance criterion for entry. If the first variable is entered, the explanatory variable not in the regression equation that has the largest partial correlation is considered next. The forward selection procedure would stop when there are no explanatory variables that meet the entry criterion. The back elimination procedure initially considers all explanatory variables to be included in the regression equation and then sequentially removed. The explanatory variable with the smallest partial correlation with the dependent variable is first for the removal. If that variable meets the tolerance criterion for elimination, it is removed. After the first variable is removed, the variable remaining in the regression equation with the smallest partial correlation is considered next. The procedure would stop when there are no variables in the regression equation that satisfy the removal criteria. At each step for the stepwise selection procedure, the explanatory variable which is not in the regression equation that has the smallest probability of *F* is entered, if the probability is sufficiently small. Explanatory variables that have already existed in the regression equation are removed if their probability of *F* is sufficiently large. The stepwise selection procedure would stop when no more variables are eligible for inclusion or removal.

In this study, all those three selection procedures resulted in the same MR model.  Table 3 lists the results of the parameter estimates. As shown in Table 3, all the values of VIFs of the remaining variables are smaller than 10. Accordingly, there is no high collinearity among these explanatory variables. This model is described in the following:

(16) Y=12.253+0.101X1−0.133X2−0.685X3+0.753X6 +0.653X12−1.971X13.

It has been reported that more than 75% of neural networks applications would employ the BPN structure, and, thus, this study uses the BPN in building the ANN forecasting model [8, 38]. Also, since one-hidden-layer network has been reported to be sufficient to model the complex system, this study considers one hidden layer for the ANN modeling structures [39–41]. This study uses 13 input nodes (or explanatory variables) and one output node. The hidden nodes range from *n* − 2 to *n* + 2, where *n* is the number of input variables. Thus, the hidden nodes are chosen as 11, 12, 13, 14, and 15, respectively. The ANN model has only one output node, the prediction of BFP. According to the findings of [42], the learning rates were set to be 0.01, 0.005, and 0.001, respectively.

In addition, since MAPE is one of the most important performance measurements for the forecasting capability, this study uses the smallest MAPE as the criterion for selecting the ANN topology. After performing the ANN modeling, this study found that the {13-11-1} topology with a learning rate of 0.01 provides the best results and a minimum testing MAPE. Here, {*n*_*i*_-*n*_*h*_-*n*_*o*_} stands for the number of neurons in the input layer, number of neurons in the hidden layer, and number of neurons in the output layer, respectively.  Table 4 presents the corresponding MAPE values for various settings of the ANN topologies. Accordingly, the ANN topology of {13-11-1} with a learning rate of 0.01 is chosen for the model of ANN alone.

This study also performed the MARS modeling to the BFP dataset; the selection results are displayed in Table 5. During the selection process, four important explanatory variables were chosen. The corresponding relative importance indicators are shown in the last column of Table 5. As a consequence, those seven important variables would be served as the input variables for intelligent hybrid modeling process. For SVR modeling, same as ANN modeling, we have 13 input variables. The two parameters, *C* and gamma, were estimated to be 2^3^ and 2^−7^, respectively.

### 3.3. Proposed Hybrid Modeling Approaches

In this study, the initial stage of the proposed hybrid modeling is to obtain the fewer but more important input variables for the second stage of forecasting models. Because this study utilizes MR and MARS modeling selections, the explanatory variables which were selected from MR and MARS models were used to serve as the input variables for other prediction models. Accordingly, this study employs six combinations of the candidate hybrid models to predict the BFP. Those hybrid models include MR-ANN, MR-MARS, MR-SVR, MARS-MR, MARS-ANN and MARS-SVR, respectively.

For the hybrid MR-ANN model, this study sets up 6 input nodes in the input layer, and the number of hidden nodes was set to 4, 5, 6, 7, and 8. The learning rates were identical to those used in the single-stage ANN model. The {6-6-1} topology with a learning rate of 0.01 provided the best results for the hybrid MR-ANN model. For the MARS-ANN hybrid model, this study used 4 input nodes in the input layer. The number of hidden nodes was set to 2, 3, 4, 5, and 6. Accordingly, the {4-3-1} topology with a learning rate of 0.01 provided the best results. For the other hybrid models, this study used 6 and 4 important explanatory variables, which are selected by using MR and MARS models, respectively, to predict BFP.

### 3.4. Prediction Results and Performance Comparison

In addition to presenting the single-stage prediction modeling, this study develops various hybrid models for prediction of BFP. This study considers the forecasting accuracy measures of MAPE, MSE, and MAD to address the forecasting performance for typical single-stage and the proposed hybrid models. The prediction measurements are defined as follows:

(17) $$\begin{array}{c} \text{MAPE}=\frac{1}{n}\sum_{t=1}^{n}\frac{\left|e_{t}\right|}{Y_{t}}\times 100,\\ \text{RMSE}=\sqrt{\frac{1}{n}\sum_{t=1}^{n}{\left(e_{t}\right)}^{2}},\\ \text{MAD}=\frac{1}{n}\sum_{t=1}^{n}\left|e_{t}\right|, \end{array}$$

where *e*_*t*_ stands for the residual at time *t*. A low MAPE, MSE, or MAD is associated with better forecasting accuracy.

The prediction results for single and the proposed hybrid modeling are listed in Table 6. In Table 6, by considering single-stage modeling approaches, we note that SVR model has the best performance in terms of MAPE, RMSE, or MAD indices. For the hybrid models, the MR-MARS has the best prediction performance. In comparison to the single-stage and the proposed hybrid models in Table 6, one is able to observe that some of our proposed hybrid models provide more accurate results than the single-stage models. For example, in terms of MAPE, MSE, or MAD, the proposed hybrid MR-MARS model possesses the lowest values among all the models.

Accordingly, the MAPE percentage improvements of the proposed MR-MARS model over the single MR and MARS models are 4.2% and 9.5%, respectively. In addition, the MAPE percentage improvements of the proposed MR-SVR model over the single MR and SVR models are 4.1% and 3.8%, respectively. Although the hybrid ANN models do not make any significant improvements, the fewer body circumference measurements were used for our proposed approach. This is another significant advantage of using our hybrid models.

## 4. Conclusion

Maintaining appropriate body fat is very crucial for human's health. However, the measurement of the BFP is not straightforward. Accordingly, this study proposes the hybrid models to effectively predict BFP. Although the 13 body circumference measurements are involved in the real dataset, the proposed models are able to provide better predictions with fewer body circumference measurements. Actually, it is much more convenient to predict BFP with fewer body circumference measurements for most of the people.

The rationale of the proposed hybrid modeling was initially to obtain fewer important explanatory variables by performing MR and MARS modeling techniques. Those important variables were served as inputs for other designed prediction models. According to the modeling results, the proposed hybrid approaches were most appropriate for predicting the BFP. Additionally, the proposed hybrid MR-MARS model was the best alternative because it contained fewer number of explanatory variables and provided the best prediction MAPE precision.

In order to obtain more accurate predictions of BFP, in addition to the existing 13 body circumference measurements, one may collect some other measurements so that the prediction precision can be increased. Moreover, the proposed hybrid modeling is not the only prediction technique that can be employed. One may combine other data mining techniques, such as rough set or genetic algorithms, to refine the structure of the hybrid models. The possibility to apply the same procedure to combine other methods as are the evolving systems deserves further research.

## Figures and Tables

**Table 1:** Variables' definition in the BFP dataset.

Variable	Meaning
*D*	Density determined from underwater weighing
*Y*	BFP
*X*_1_	Age (years)
*X*_2_	Height (cm)
*X*_3_	Weight (kg)
*X*_4_	Neck circumference (cm)
*X*_5_	Chest circumference (cm)
*X*_6_	Abdomen 2 circumference (cm)
*X*_7_	Hip circumference (cm)
*X*_8_	Thigh circumference (cm)
*X*_9_	Knee circumference (cm)
*X*_10_	Ankle circumference (cm)
*X*_11_	Biceps (extended) circumference (cm)
*X*_12_	Forearm circumference (cm)
*X*_13_	Wrist circumference (cm)

**Table 2:** Pearson correlations for pairs of variables.

	*Y*	*X*_1_	*X*_2_	*X*_3_	*X*_4_	*X*_5_	*X*_6_	*X*_7_	*X*_8_	*X*_9_	*X*_10_	*X*_11_	*X*_12_	*X*_13_
*Y*	1.00													
*X*_1_	0.296	1.00												
*X*_2_	−0.076	−0.243	1.00											
*X*_3_	0.559	−0.065	0.454	1.00										
*X*_4_	0.433	0.069	0.275	0.820	1.00									
*X*_5_	0.674	0.091	0.196	0.890	0.770	1.00								
*X*_6_	0.789	0.180	0.169	0.882	0.735	0.907	1.00							
*X*_7_	0.568	−0.091	0.323	0.944	0.736	0.841	0.876	1.00						
*X*_8_	0.538	−0.210	0.244	0.872	0.706	0.759	0.788	0.898	1.00					
*X*_9_	0.492	−0.043	0.421	0.854	0.660	0.727	0.756	0.831	0.814	1.00				
*X*_10_	0.236	−0.092	0.356	0.586	0.435	0.463	0.429	0.533	0.489	0.580	1.00			
*X*_11_	0.447	−0.049	0.261	0.784	0.701	0.718	0.667	0.732	0.764	0.678	0.432	1.00		
*X*_12_	0.317	−0.127	0.272	0.597	0.587	0.543	0.456	0.497	0.543	0.501	0.358	0.634	1.00	
*X*_13_	0.308	0.152	0.364	0.737	0.732	0.655	0.614	0.646	0.585	0.645	0.551	0.630	0.564	1.00

**Table 3:** The results of parameter estimates using three selection procedures.

Variables	Coefficient estimates	*t*	VIF
Constant	12.253	1.247	
*X*_1_	0.101	3.583	1.250
*X*_2_	−0.133	−2.408	1.292
*X*_4_	−0.685	−2.971	3.244
*X*_6_	0.753	16.479	2.317
*X*_12_	0.653	3.143	1.739
*X*_13_	−1.971	−3.509	2.662

**Table 4:** The topology setting results for the ANN model alone.

ANN topology	MAPE
{13-11-1}	25.33694
{13-12-1}	25.48976
{13-13-1}	25.39417
{13-14-1}	25.49184
{13-15-1}	25.51463

**Table 5:** Basis functions and important explanatory variables for the MARS model.

Function	Std. dev.	Cost of omission	Number of BF	Variable	Relative importance (%)
1	8.870	33.093	2	*X*_6_	100.000
2	2.000	19.513	1	*X*_8_	38.199
3	2.021	18.448	1	*X*_13_	28.095
4	2.865	17.664	1	*X*_3_	17.210
5	1.132	17.652	1	*X*_1_	16.981
6	1.230	17.627	1	*X*_12_	16.511
7	1.099	17.506	1	*X*_4_	14.027

**Table 6:** Performance comparison of typical single-stage and the proposed hybrid models.

	MAPE	RMSE	MAD
Single-stage models			
MR	26.8337	4.8033	3.9596
ANN	25.3369	4.6958	3.7805
MARS	25.3455	4.6498	3.7646
SVR	25.2577	4.6432	3.7568
Proposed hybrid models			
MR-ANN	25.7275	4.7099	3.8169
MR-MARS	24.2874	4.6384	3.6974
MR-SVR	25.2996	4.6427	3.8402
MARS-MR	25.8319	4.6760	3.8636
MARS-ANN	25.8000	4.6631	3.8898
MARS-SVR	24.3078	4.6946	3.8967
